# Identification and Determination of the Polyhydroxylated Alkaloids Compounds with α-Glucosidase Inhibitor Activity in Mulberry Leaves of Different Origins

**DOI:** 10.3390/molecules21020206

**Published:** 2016-02-08

**Authors:** Tao Ji, Jun Li, Shu-Lan Su, Zhen-Hua Zhu, Sheng Guo, Da-Wei Qian, Jin-Ao Duan

**Affiliations:** Jiangsu Collaborative Innovation Center of Chinese Medicinal Resources Industrialization, Nanjing University of Chinese Medicine, Nanjing 210023, China; nianningtao@163.com (T.J.); justleejun@sina.com (J.L.); 04040416@163.com (Z.-H.Z.); gsh916@163.com (S.G.); qiandwnj@126.com (D.-W.Q.)

**Keywords:** 1-deoxynojirimycin, cluster analysis, HPLC-ELSD, mulberry leaves, quality control

## Abstract

Mulberry leaves have commonly been utilized in China as a herbal medicine for the treatment of diabetes for thousands of years. To evaluate the quality, an ultra-high performance liquid chromatography coupled with quadrupole time of flight mass spectrometry (UPLC-Q-TOF/MS) method was developed for identification of polyhydroxylated alkaloids with α-glucosidase inhibitor activity in mulberry leaf. As a result, five alkaloid compounds were identified or tentatively characterized. Among them, the compound 1-deoxynojirimycin (DNJ) was selected as the most typical and active chemical marker and quantified using an improved high performance liquid chromatography (HPLC) normal phase coupled with evaporative light scattering detector (ELSD) method. The developed method was fully validated in terms of linearity, sensitivity, precision and repeatability, as well as recovery, and subsequently applied to evaluate twenty-nine batches of mulberry leaves from different collections. From the analytical data it was discovered that the average content of DNJ is 1.53 mg/g, while the total contents of DNJ in the 29 mulberry leaf sample ranged from 0.20 to 3.88 mg/g, which suggested remarkable differences, although it reached the highest levels in early August. These data may provide an important reference for the quality of mulberry leaves used as herbal medicine for the treatment of diabetes or as a material to obtain the DNJ of α-glucosidase inhibitor or as a functional food.

## 1. Introduction

*Morus alba* L., which is a Moraceous plant, has a history of over 4000 years in China. The leaves of mulberry tree are considered as the food of silkworms and have been commonly used as a food, food additive and folklore medicine. There are 15 kinds of mulberry, which are widely distributed all over the country. The leaves and root bark of the mulberry tree are known worldwide as sources of phytotherapeutics, which have traditionally been used for the treatment of conditions related to type II diabetes [1,2,3,4,5]. A large number of *in vivo* animal and human studies support the fact that the leaves and roots of mulberry tree have been commonly used as a traditional Chinese medicine for their hypolipidemic [6,7], antihypoglycemic [8,9,10], antioxidant [11,12,13], antihypertensive [14], anti-inflammatory [15,16], anti-atherosclerotic [17], antitumor [18], anticonvulsant [19] and vasodilator [20] effects. Phytochemical studies showed that mulberry leaves are rich in a variety of constituents, including flavonoids [21,22,23], alkaloids [24,25], polysaccharides [26], amino acids, nucleosides [27], fatty acids [28], organic acid [29,30], microelements, and so on.

**Figure 1:**
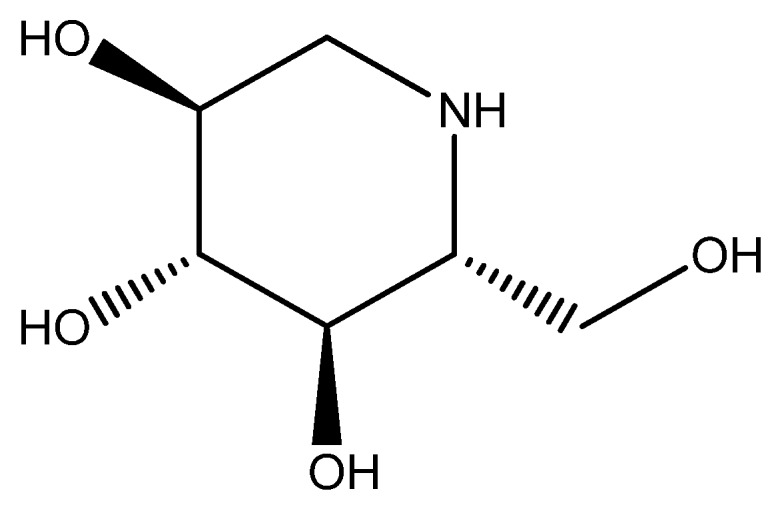
The chemical structure of DNJ.

1-Deoxynojirimycin (DNJ), which is a main active constituent in mulberry leaf, has attracted remarkable interest because of its effective and specific inhibition of different carbohydrate-degrading enzymes involved in a wide range of important biological processes, including hepatic glycogen breakdown, lysosomal catabolism of glycoconjugates, intestinal digestion, and maturation of the sugar chains in glycoproteins [31]. What is well known that DNJ is an α-glucosidase inhibitor [32,33], which acts as an antihyperglycemic agent by slowing the rate of carbohydrate degradation to monosaccharides, and it can delay glucose absorption and significantly reduce post prandial blood glucose levels [34]. The structure of 1-deoxynojirimycin is similar to that of sugars (Figure 1).

Like many other aminoglycosides, DNJ lacks a suitable chromophore, which is necessary for UV detection. It has been reported that analysis of DNJ could be performed using ultra-high performance liquid chromatography with triple quadrupole tandem mass spectrometry [35,36]. Although this method shows great advantages over traditional HPLC for its high power in separation, analysis speed and high sensitivity, triple quadrupole mass detection and multiple reaction monitoring is difficult to perform by laboratories with little experience, and the Direct Analysis in Real Time (DART) ionization source coupled with triple quadrupole tandem mass spectrometry method has the same problem [37]. Pre-column derivatization methods are also an common choice [38,39], but they require cumbersome steps and considerable sample manipulation which makes the HPLC analysis more complicated and time-consuming. Besides, derivatization methods may result in introduction of non-controlled impurities and degradation products. Another method involving GC-MS analysis with derivatization has similar problems [40]. Moreover, the GC-MS instruments tend to be more expensive, complicated, and it the method has the fatal disadvantage of requiring the removal of water from samples prior to silylation.

The evaporative light scattering detector (ELSD) has been proven to be a promising technique for analyses of solids and liquids samples with minimal or no sample preparation. It is a “universal” detector for relatively non-volatile analytes without the need for derivatization. Compared with the RI detector, the ELSD has a higher sensitivity and is much more economical than MS. To our knowledge, it has been reported that Kimura *et al*. [41] successfully separated DNJ from an extract of mulberry leaves on a TSKgel Amide-80 column using HPLC-ELSD, but the method required a long time to complete the analysis.

The aim of this work was to identify the polyhydroxylated alkaloid compounds in mulberry leaves using UPLC-QTOF/MS and propose a more rapid, reliable and convenient HPLC-ELSD method for the determination of DNJ, the most typical naturally occurring alkaloid with promising biological activity in *M. alba* leaves, by choosing a different column and comparing the mobile phase systems and ELSD parameters. Then the improved method was applied for the analysis of 29 batches of mulberry leaf samples in order to provide a foundation for the quality control of mulberry leaf herbs and various food products containing mulberry leaves, which can ensure the authenticity and contents of DNJ in these products and verify the label claims. Furthermore, clustering analysis was adopted to study the characteristics of different kinds of samples in order to evaluate the quality of the mulberry leaves and provide a reference for studying the plant species relationships among the samples.

## 2. Results and Discussion

### 2.1. Identification of Polyhydroxylated Alkaloids in the Samples by UPLC-QTOF/MS

**Figure 2:**
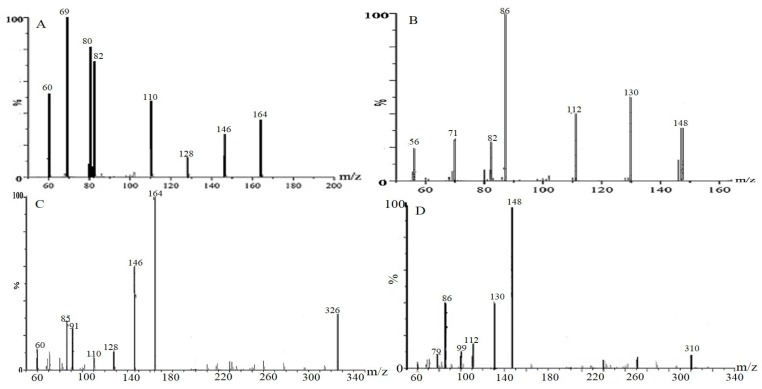
The secondary mass spectrograms of four *M. alba* alkaloid compounds: (**A**) DNJ; (**B**) Fagomine; (**C**) 2-*O*-α-d-Gal-DNJ; (**D**) 4-*O*-β-d-Glc-fagomine.

Under the used chromatographic and MS conditions, five peaks were detected in all 29 samples. DNJ and fagomine were identified by comparison of their HPLC retention times and ESI-MS/MS spectrometric data with those of reference compounds. Owing to the unavailability of authentic standards, three peaks were tentatively assigned as isofagomine, 2-*O*-α-d-Gal-DNJ and 4-*O*-β-d-Glc-fagomine by comparing the ESI-MS/MS spectrometric data with the published research about the components in *Morus alba* L. [42,43]. The results are summarized in Table 1 and Figure 2. In the MS spectra, the most prominent mass-to-charge ratios corresponded to the protonated molecular ions of the five compounds. Furthermore, fragments corresponding to the loss of the sugar moieties for compounds **4**,**5** were found in the tandem mass spectrometry (MS/MS) traces. All the fragment ions mentioned above were consistent with those of reference standards, which further confirmed our identification of the constituents in mulberry leaves.

**Table 1:** Spectrometric data of compounds found in mulberry leaves.

No.	M (*m*/*z*)	Measured/[M + H]^+^ (*m*/*z*)	*m*/*z* (MS/MS)	Formula	Compound
1	163.08	164.17	146, 110, 82	C_6_H_13_NO_4_	DNJ
2	147.17	148.18	112, 86, 56	C_6_H_13_NO_3_	Fagomine
3	147.17	148.18	112, 84, 56	C_6_H_13_NO_3_	Isofagomine
4	325.24	326.31	164, 146, 110, 85	C_12_H_23_NO_9_	2-*O*-α-d-Gal-DNJ
5	309.33	310.32	148, 112, 86	C_12_H_23_NO_8_	4-*O*-β-d-Glc-fagomine

### 2.2. Optimization of the HPLC Separation

In our preliminary tests, we chose an X Bridge amide 3.5 μm column (150 mm × 4.6 mm) to separate DNJ from other compounds. As for the mobile phase, we compared methanol and acetonitrile for their performance in separating mulberry leaf extract. Methanol is a polar protic solvent, while acetonitrile belongs to the polar aprotic solvent class, which probably can explain why acetonitrile showed more powerful separation ability for the samples. As is known to all, acids and alkalis can improve HPLC peak shapes. Besides, it has been reported that aqueous ammonium acetate or ammonium formate solution that can also improve the separation of alkaloids for HPLC analysis, so we compared the separation effect of several different mobile phase additives, including formic acid, ammonia, ammonium formate and ammonium acetate. The results showed that acetate used as a salt additive to the mobile phase could provide much improved sensitivity and peak shapes. Next different concentrations of ammonium acetate (4.5, 5.5, 6.5, 7.5 mmol/L) was compared. The result showed that the solution at 6.5 mmol/L was better than those at 4.5, 5.5 and 7.5 mmol/L for improving the peak shape. As for the type of mobile phase, both gradient and isocratic elution have been used in the tests. The results revealed that the latter provided a better resolution when retention time is taken into account. After comparing different mobile phase proportions, 70% acetonitrile was applied. The injection volume was set at 2 μL while the flow rate was set at 0.8 mL/min and the column temperature was kept at 30 °C.

### 2.3. Optimization of ELSD Conditions

**Figure 3:**
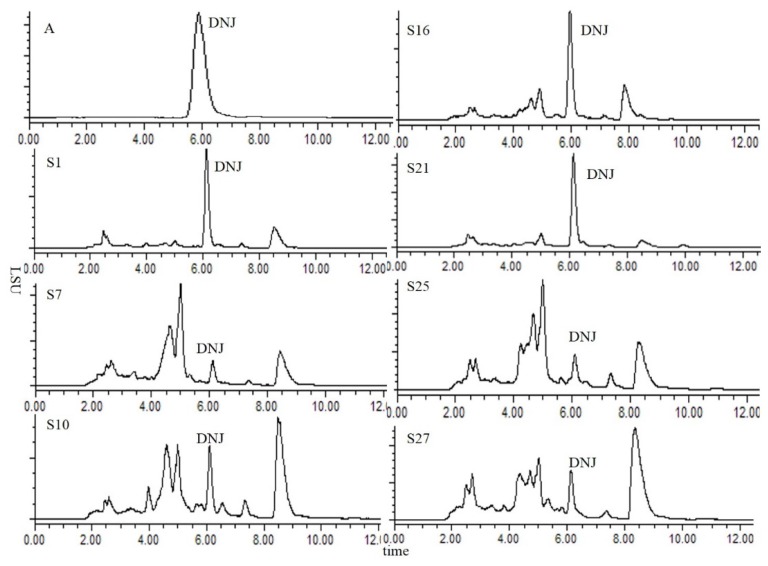
HPLC-ELSD chromatogram of DNJ. (**A**) DNJ standard chromatogram; the others are DNJ chromatograms of samples.

A Waters 2424 ELSD (Milford, MA, USA) was used in the analysis and it was recommended that its drift tube temperature be set at 55 °C. Based on this, temperatures of 50, 60, 65, 70 and 75 °C were compared to study the ELSD sample peak area response while the air flow rate was fixed at 2.7 L/min. The results suggested that 70 °C was optimal. The mode of nebulizing air was heating and the degree of power was 60%. Representative HPLC-ELSD chromatograms of DNJ and part of the samples separated under the optimized chromatography and detection conditions are shown in Figure 3.

### 2.4. Analytical Method Validation

The method validation assays were carried out under the optimized conditions, and included the linearity, LOD, LOQ, precision, repeatability, stability, and recovery. The linearity was determined by six concentration levels of DNJ ranging from 0.025 to 1.02 mg/mL. The results showed that the calibration curves exhibited excellent linear regressions with a high determination coefficient (*r*^2^ = 0.997) and the calibration range adequately covered the amounts of DNJ in the investigated samples. The LOD and LOQ were found to be 6.8 μg/mL and 20.3 μg/mL. The precision, repeatability, recovery, and stability RSD were all ≤2.3%, which demonstrated that the established method was accurate enough for determination of DNJ and the samples are suitable for analysis. All the data are shown in Table 2.

**Table 2:** Characteristics of chromatographic peaks of DNJ in method validation.

No.	Item	DNJ
1	Calibration curves	*y* = 1.394*x* + 11.54
2	Linear range (mg/mL)	0.025~1.015
3	Correlation coefficient, *r*^2^	0.997
4	Precision RSD, %, *n* = 6	1.1
5	Repeatablity RSD, %, *n* = 6	1.8
6	Stability RSD, %, *n* = 6	1.4
7	Recovery, Mean, RSD% (*n* = 3) Low level (80%) Medium level (100%) High level (120%)	94.78, 2.95 95.21, 3.01 94.96, 3.12
8	Retention time, min	5.8
9	LOD (μg/mL)	6.8
10	LOQ (μg/mL)	20.3

### 2.5. Quantification of DNJ in 29 Batches of Mulberry Leaves Samples

The contents of DNJ in 29 batches of mulberry leaves samples were quantified according to the established HPLC-ELSD method. The results showed that all of these mulberry leaf samples were rich in DNJ, although their contents were obviously different. The average content of DNJ was 1.53 mg/g, while the total contents of DNJ in the 29 mulberry leaves samples ranged from 0.20 to 3.88 mg/g, which suggested remarkable differences. The data showed that the content of DNJ in mulberry leaves which were collected in the same place changed with time. For samples collected from Bozhou, the DNJ content reached its highest value in early August. In the point of origin analysis, Jiangsu Province reached the highest level, whereas it was only 0.20 mg/g in sample 12, which was collected in Bozhou, Anhui Province. The data is listed in Table 3. These data provide an important reference for the quality of mulberry leaves used as herbal medicine for the treatment of diabetes or as a material to obtain the DNJ for use as an α-glucosidase inhibitor or as a functional food.

**Table 3:** Contents of DNJ in 29 batches of mulberry leaves samples (*n* = 3).

Sample	Origin	Batch Number	Content (mg/g)	RSD (%)
S1	Xinjang	130408	2.07	0.12
S2	Xinjang	131102	0.78	0.11
S3	Xinjang	131128	0.95	0.18
S4	Hangzhou, Zhejiang	141106	1.72	0.11
S5	Hangzhou, Zhejiang	141102	2.05	0.09
S6	Dabieshan, Anhui	140901	1.38	0.07
S7	Dabieshan, Anhui	141006	1.24	0.14
S8	Bozhou, Anhui	130101	1.72	0.12
S9	Bozhou, Anhui	140616	0.82	0.09
S10	Bozhou, Anhui	140805	1.84	0.13
S11	Bozhou, Anhui	140815	1.55	0.19
S12	Bozhou, Anhui	140905	0.20	0.11
S13	Bozhou, Anhui	141006	0.28	0.09
S14	Tongling, Anhui	140301	1.02	0.14
S15	Hefei, Anhui	130111	1.11	0.17
S16	Nanjing, Jiangsu	141022	0.79	0.12
S17	Nanjing, Jiangsu	141025	1.95	0.11
S18	Yixing, Jiangsu	141015	1.11	0.15
S19	Wuxi, Jiangsu	141015	1.43	0.14
S20	Zhenjiang, Jiangsu	141120	3.15	0.11
S21	Y64, Zhenjiang	121101	3.88	0.16
S22	Y66, Zhenjiang	121101	3.01	0.14
S23	Nantong, Jiangsu	140920	0.62	0.18
S24	Wuhan, Hubei	140902	0.57	0.07
S25	Tongrentang, Nanjign	120804	1.95	0.15
S26	Xuandetang, Nanjing	120813	1.17	0.14
S27	Xiansheng, Nanjing	130130	1.99	0.16
S28	Bailong, Nanjing	130303	2.31	0.11
S29	Yifeng, Nanjing	130211	1.35	0.13

### 2.6. The Clustering Analysis of 29 Batches of Mulberry Leaves Samples

**Figure 4:**
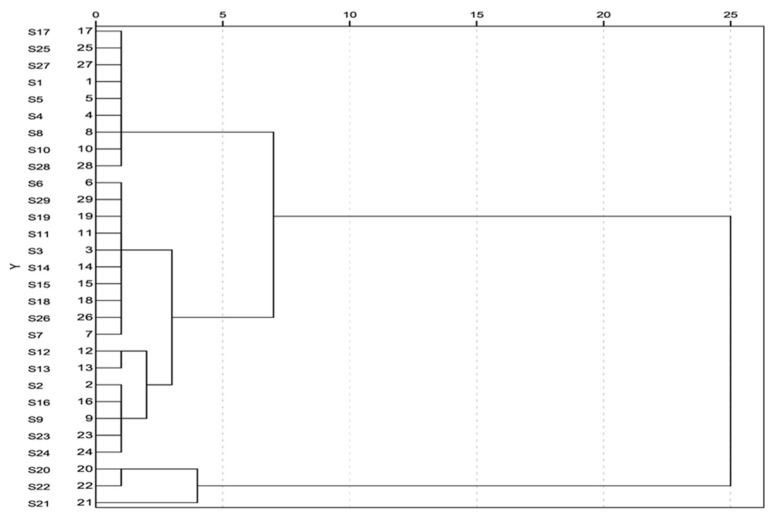
Dendrogram using Average Linkage (between Groups) Rescale Distance Cluster Combine.

A dendrogram, which lists all the samples, was constructed to present the relationship between the DNJ contents and the origins of the mulberry leaves based on cluster analysis. Based on this dendrogram, shown in Figure 4, the samples were grouped in three well separated groups with a number of clusters of 5. Samples S17, S25, S27, S1, S5, S4, S8, S10, and S28 were the most similar and together formed the first cluster, samples S20 and S22 were also very similar and were combined to form the next cluster, followed by the addition of sample S21. Samples S12 and S13 were the third cluster, followed by the joining of samples S2, S16, S9, S23 and S24 at similar levels, as did the remaining samples. While the number of clusters ranged from 7 to 25, the samples have been divided into two groups. Interestingly, the samples S20, S22 and S21 grouped independently, even though the number of clusters was changed. The data implied that the mulberry leaf samples collected in Zhenjiang could be of authentic origin due to their high content of DNJ, especially the Y64 mulberry leaves. Besides, the samples from Xinjiang and Zhejiang provinces were also the key sources of DNJ. The difference of content between the samples may be caused by the different species, geographical environment and picking period.

## 3. Experimental Section

### 3.1. Chemicals and Reagents

Acetonitrile was of HPLC grade (Tedia, Fairfield, OH, USA), deionized water (H_2_O) was purified by a super purification system (Eped Technology Development, Nanjing, China),and formic acid was of HPLC grade (Merck, Darmstadt, Germany).Other reagent solutions, such as ammonia (Qingzhou Lutai Chemical Reagent, Qingzhou, China)and ammonium acetate (Sinopharm Chemical Reagent, Shanghai, China), were of analytical grade. The chemical standard of 1-deoxynojirimycin (DNJ) was purchased from the National Institute for the Control of Pharmaceutical and Biological Products (Beijing, China). The purity of the DNJ was >98%, as determined by HPLC analysis.

### 3.2. Plant Materials

Twenty-four batches of mulberry leaves samples (samples S1–S24) were collected on different dates from four provinces, including Jiangsu, Zhejiang, Xinjiang and Anhui. Five batches of mulberry leaves samples (samples S25–S29) were purchased from five traditional medicine pharmacies in Nanjing (China). After collection, the leaves were dried at 50 °C in an oven. The different batches of mulberry leaves powder showed very different appearances. The color of the majority of samples was green, and after the frost this turns to yellow. Their botanical origins were identified by the second corresponding author, and the voucher specimens were deposited at the Herbarium in Jiangsu Key Laboratory for High Technology Research of TCM Formulae, Nanjing University of Chinese Medicine (Nanjing, China).

### 3.3. Preparation of Sample Solutions

The dried mulberry leaves were pulverized to homogeneous powders (40 mesh). An aliquot of dried powder (5.0 g) was weighed accurately into a 1000 mL round bottom flask and 200 mL 70% ethanol was added. After heating to reflux twice for 45 min, then the extracting solution was filtered. After merging the two filtrates and concentrating to 50 mL using a rotary evaporator, the supernatant was stored on the sample plate whose temperature was set at 4 °C before injection into the HPLC system for analysis.

### 3.4. UPLC-QTOF/MS Conditions for Identification Analyses

The identification of polyhydroxylated alkaloids was performed on a quadrupole time of flight mass spectrometer connected to a Water ACQUITY UPLC system (Waters Corp., Milford, MA, USA) via an electrospray ionization interface (ESI) source. An Acquity UPLC BEH Amide (100 mm × 2.1 mm, 1.7 μm) column was applied for identification analyses. The mobile phase was composed of A (Water containing ammonia; pH 8.0) and B (acetonitrile) and a gradient elution profile was used: 0~3 min, 5%~50%A; 3~8 min, 50%A; 8~9 min, 50%–5%A; 9~10 min, 5% A. The flow rate of the mobile phase was 0.4 mL/min, and the column temperature was maintained at 35 °C. All the data were acquired and analyzed by MassLynx Software Version 4.1 (Waters Corp., Milford, MA, USA). The Q/TOF Mass spectrometer was operated in positive ion mode with a capillary voltage of 3 kV, sampling cone voltage of 20 eV, cone gas flow of 50 L/h, desolvation gas flow of 1000 L/h, desolvation temperature of 550 °C, source temperature of 150 °C, collision energy of 22 eV, and the full scan spectra from 100 to 1000 Da.

### 3.5. Preparation of Standard Solutions

DNJ standard stock solution of 1.02 mg/mL was prepared by accurately weighing and dissolving the compound in water. The stock standard solution is stable when stored in a refrigerator at 4 °C. Then the stock standard solution was serially diluted with water in order to prepare calibration standard solutions of 1.02, 0.5, 0.25, 0.1, 0.05 and 0.025 mg/mL, respectively. A 2 μL sample was injected into HPLC-ELSD each time. The external standard curve was made by making the best fit of the equation *y* = a*x* + b (*y* represented the logarithmic value of the peak area response and *x* represented the value of the concentration of DNJ (μg/mL) using least-squares linear regression.

### 3.6. Chromatographic Conditions and Instrument for Quantitative Analyses of DNJ

The analysis was performed on a Waters 2695 Alliance HPLC system (Waters, Milford, MA, USA), with a Waters 2424 ELSD coupled to the HPLC system. Compressed air (from a XWK-3A air pump, Tianjin Huasheng Analytical Instrument, Tianjin, China) was used as the nebulizing gas for ELSD. Chromatographic data were collected and processed by an Empower™ chromatographic working station (Waters).The separation was conducted on an XBridge amide 3.5 μm column with dimensions of 150 mm × 4.6 mm (Waters). An isocratic separation method was developed and optimized by testing different mobile phase system, concentrations of ACN and water solvents (ammonia, ammonium acetate or formic acid added in water) and ELSD parameters, including the temperature of the ELSD drift tube, the carrier gas pressure and the air nebulizing gas flow rate.

### 3.7. Validation of the Method

For calibration, the linearity was diluted with water to a series of appropriate concentrations and obtained by plotting the logarithmic of peak areas *versus* the corresponding concentrations. The signal-to-noise (S/N) ratio of 3 was selected for the LOD and the S/N of 10 for LOQ. The precision was evaluated by analyzing the standard solutions six times. Variations of the peak area were taken as the measures of precision and expressed as percentage relative standard deviations (RSD). Six independent analytical sample solutions prepared from the same sample were analyzed to evaluate the repeatability, RSD represented the variations. To confirm the stability of the solution, one of the sample solutions mentioned above was stored at 25 °C and analyzed at 0, 2, 4, 8, 12, and 24 h, respectively. A recovery test was performed by adding corresponding marker compounds with high (120%), medium (100%), and low (80%) levels into accurately weighed samples to evaluate the accuracy of this method.

### 3.8. Cluster Analysis

In order to study the relationship between the contents of DNJ and the origins of mulberry leaf samples, cluster analysis was applied with the help of the SPSS 16.0 software (SPSS Inc, Chicago, IL, USA). For cluster analysis, the DNJ contents of all samples were imported into the SPSS software to construct the dendrogram by means of the clustering method of Between-groups linkage (Hierarchical Cluster Analysis) using squared Euclidean distance as the distance measurement method. The results were presented on a dendrogram, so the more close the DNJ content of two samples, the less the relative distance between them on the dendrogram.

## 4. Conclusions

In this study, five polyhydroxylated alkaloid compounds were identified or tentatively characterized, and DNJ was selected as the most typical and active chemical marker and quantified within 15 min using an improved HPLC coupled with ELSD detector method without derivatization. After validating for good precision, repeatability, stability and recovery, the method was proved an efficient alternative choice for determination of DNJ in mulberry leaves, which is simple and direct compared with other methods and the application of the method to twenty-nine batches of leaf samples allowed us to conclude that it could serve as a prerequisite for the quality control and standardization of mulberry leaves and various food products containing mulberry leaves. The contents of DNJ in the 29 samples demonstrated an origin difference and the clustering analysis results showed that all the samples can be divided into two groups while the number of clusters was set at 7. The sample S20, S21 and S22, which were all collected from mulberry garden in Zhenjiang, Jiangsu Province, could be a promising natural sources for future industrial research of DNJ due to their high DNJ content that could bring in a lot of potential human health benefits. Meanwhile, it provides a good basis for picking the mulberry leaves with abundant DNJ and discussing the plant species relationships among different samples.
